# RNA-binding protein SAMD4 regulates skeleton development through translational inhibition of Mig6 expression

**DOI:** 10.1038/celldisc.2016.50

**Published:** 2017-01-24

**Authors:** Ningning Niu, Jian-Feng Xiang, Qin Yang, Lijun Wang, Zhanying Wei, Ling-Ling Chen, Li Yang, Weiguo Zou

**Affiliations:** 1 State Key Laboratory of Cell Biology, CAS Center for Excellence in Molecular Cell Sciences, Institute of Biochemistry and Cell Biology, Chinese Academy of Sciences, Shanghai, China; 2 State Key Laboratory of Molecular Biology, CAS Center for Excellence in Molecular Cell Sciences, Institute of Biochemistry and Cell Biology, Chinese Academy of Sciences, Shanghai, China; 3 Key Laboratory of Computational Biology, CAS-MPG Partner Institute for Computational Biology, Shanghai Institutes for Biological Sciences, Chinese Academy of Sciences, Shanghai, China; 4 Metabolic Bone Disease and Genetic Research Unit, Division of Osteoporosis and Bone Disease, Department of Endocrinology and Metabolism, Shanghai Jiao Tong University Affiliated Six People’s Hospital, Shanghai, China

**Keywords:** bone development, chondrocyte, Mig6, osteoblast, SAMD4, translational repression

## Abstract

Protein translation regulation has essential roles in inflammatory responses, cancer initiation and the pathogenesis of several neurodegenerative disorders. However, the role of the regulation of protein translation in mammalian skeleton development has been rarely elaborated. Here we report that the lack of the RNA-binding protein sterile alpha motif domain containing protein 4 (SAMD4) resulted in multiple developmental defects in mice, including delayed bone development and decreased osteogenesis. Samd4-deficient mesenchymal progenitors exhibit impaired osteoblast differentiation and function. Mechanism study demonstrates that SAMD4 binds the Mig6 mRNA and inhibits MIG6 protein synthesis. Consistent with this, Samd4-deficient cells have increased MIG6 protein level and knockdown of Mig6 rescues the impaired osteogenesis in Samd4-deficient cells. Furthermore, Samd4-deficient mice also display chondrocyte defects, which is consistent with the regulation of MIG6 protein level by SAMD4. These findings define SAMD4 as a previously unreported key regulator of osteoblastogenesis and bone development, implying that regulation of protein translation is an important mechanism governing skeletogenesis and that control of protein translation could have therapeutic potential in metabolic bone diseases, such as osteoporosis.

## Introduction

Protein translational regulation of gene expression is necessary for the ability of cells to rapidly respond to the changes in the environment [1], especially in response to stress-causing stimuli, such as ultraviolet irradiation, temperature changes, nutrient limitation, oxidative stress and various drugs or toxins [2, 3]. Protein translational control is also involved in cancer development and progression, related to tumor cell survival, angiogenesis, transformation, invasion and metastasis [4, 5]. Translational regulation is also involved in mammalian development, for example, in the muscle [6], neuron [7] and early embryo [8]. However, the function of protein synthesis regulation in mammalian skeleton development remains elusive.

Skeleton development and remodeling are critical for maintenance of the biomechanical properties of bone [9]. The long bones are mainly formed through endochondral bone formation and are maintained by bone remodeling [9]. During endochondral bone formation, the chondrocytes of the cartilage template proliferate axially and subsequently undergo hypertrophy and expansion in cellular volume [10]. Osteoblasts, a group of specialized mesenchymal cells, are crucial for the mineralization of the embryonic skeleton and bone mass maintenance during postnatal bone remodeling [11, 12]. Defects in osteoblast function are a major cause of reduction in bone density, as is seen in osteoporosis, the most common skeletal disease worldwide [13, 14]. Several genes, including different transcription factors [15–17], kinases [18], ubiquitin E3 ligases [19, 20] and miRNAs [21], have been reported to have important roles during bone development. However, little is known about whether protein translational regulation acts on this process.

To elucidate the role of protein translation regulation in bone development, we conducted an unbiased forward genetic screen of a library of mutant mouse lines, which included around 4 000 different genetically manipulated lines constructed by using a *piggyBac* (PB) transposon system to mediate germline mutagenesis [22]. In screening this library for skeletal phenotypes, we identified sterile alpha motif domain containing 4 (Samd4)-deficient mice strain displaying striking lean body and narrowed thoracic cavity. SAMD4 is a mammalian homolog of *Drosophila* SMAUG protein, which has been demonstrated as a protein translational repressor [23]. The *Drosophila* SMAUG protein has been linked *in vivo* to *Drosophila* maternal mRNA destabilization [24], the maternal-to-zygotic transition [25] and early embryo development [26, 27]. The Smaug protein contains a sterile alpha motif (SAM), which directly binds RNA with stem-loop structures known as Smaug recognition elements (SREs) that contain the consensus sequence CNGG or CNGGN on target mRNAs [28]. At the same time, Smaug recruits various proteins, such as CUP [29], Argonaute 1 [30] and CUG triplet repeat RNA binding protein 1 (CUBP1) [31], to target mRNA for translational repression and/or transcript decay. Mammalian SAMD4 has been reported to be a translational repressor by *in vitro* translation assays using luciferase carrying SRE motifs [32]. However, the *in vivo* function of mammalian SAMD4 involves RNA binding and translational repression remains to be clarified.

In this study, we demonstrated that *Samd4*^*PB/PB*^ mice exhibited markedly defects in skeleton development and bone mass, along with impaired osteoblastogenesis and chondrogenesis. Further mechanism study displayed that SAMD4 binds to mitogen-inducible gene 6 (Mig6, also annotated as ERBB receptor feedback inhibitor 1, Errfi1) mRNA and repressed its translation. MIG6 is a non-kinase scaffolding adaptor [33, 34] that is highly expressed in both chondrocytes and osteoblasts [35]. Furthermore, Mig6 deficiency in mice lead to excessive articular chondrocyte proliferation following an osteoarthritis-like disorder [35, 36]. The higher MIG6 protein level in *Samd4*^*PB/PB*^ mice resulted in impaired skeletogenesis. These observations demonstrated that *in vivo* function of SAMD4 was related to protein translational regulation and suggested that SAMD4 was a novel skeletogenesis regulator. To our knowledge, this is the first report about the protein translation in the anabolic bone formation and indicates that the control of protein translation could have therapeutic potential in metabolic bone diseases, such as osteoporosis.

## Results

### The construction and identification of Samd4-deficient mice

**Figure 1:**
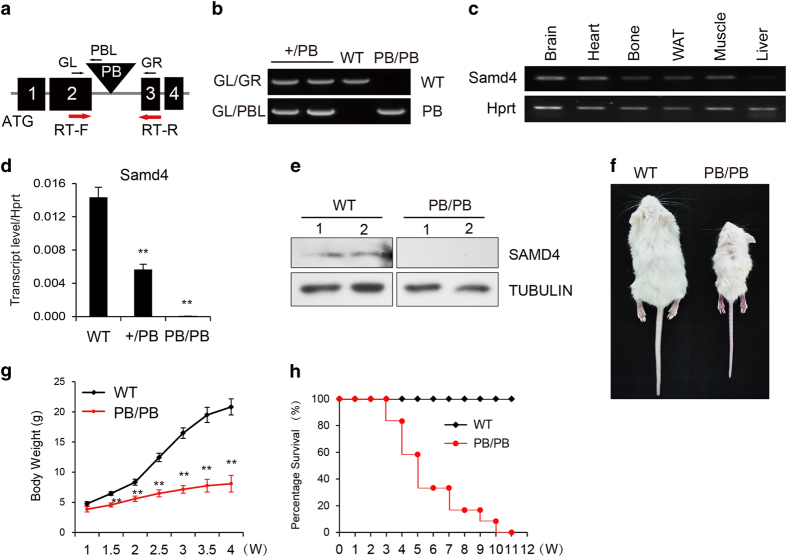
Identification and phenotypic analysis of *Samd4*^*PB/PB*^ mice. (**a**) A schematic representation of the position of *piggyBac* (PB) transposon insertion into the *Samd4* (sterile alpha motif domain containing protein 4) locus. The primers RT-F and RT-R were used for quantitative PCR (qPCR); and GL, GR and PBL were used for genotyping. Numbers 1–4 indicated exons 1–4. (**b**) Genotyping of littermates from two heterozygote cross by PCR. +/PB, the heterozygote mouse. (**c**) The reverse transcriptase-PCR analysis, which showed that Samd4 is widely expressed in the brain, heart, bone, white adipocyte and muscle, but little in the liver. (**d**) Analysis of Samd4 transcript levels by qPCR in wild-type mice (WT), *Samd4*^*+/PB*^ and *Samd4*^*PB/PB*^ tibia. (**e**) Western blotting analysis showing that Samd4 protein is absent in the protein from *Samd4*^*PB/PB*^ tibia. (**f**) Photograph of 3-week-old male WT and *Samd4*^*PB/PB*^ mouse. (**g**) Body weight of male WT and *Samd4*^*PB/PB*^ mice maintained from 1 to 12 weeks (*n*=21 for WT mice, *n*=17 for *Samd4*^*PB/PB*^ mice). (**h**) Kaplan–Meier survival curve (*n*=32 for WT mice, 24 for *Samd4*^*PB/PB*^ mice). Hprt, hypoxanthine guanine phosphoribosyl transferase; Tubulin, α Tubulin. Values represent means±s.d. *P*-values were obtained from *t*-tests with paired or unpaired samples, **P<0.01.

We screened a mutant mouse library that was constructed using a PB transposon system to mediate germline mutagenesis [22]. We observed a mouse strain displaying striking lean body and narrowed thoracic cavity. The sequencing analysis indicated that PB transposon was inserted into *Samd4* locus (Figure 1a and b). Samd4 is widely expressed in different tissues (Figure 1c), and the insertion of PB transposon at *Samd4* locus resulted in a dramatic reduction of *Samd4* transcript and protein level in mice homozygous for the *PB* transposon allele (*Samd4*^*PB/PB*^; Figure 1d and e). Analysis of littermates born to male and female mice heterozygous for the Samd4 *PB* allele (*Samd4*^*PB/+*^) revealed that *Samd4*^*PB/PB*^ mice exhibited a large reduction in body size and weight (Figure 1f and g) and had a shortened lifespan when compared with age- and sex-matched wild-type (WT) littermates (Figure 1h).

**Figure 2:**
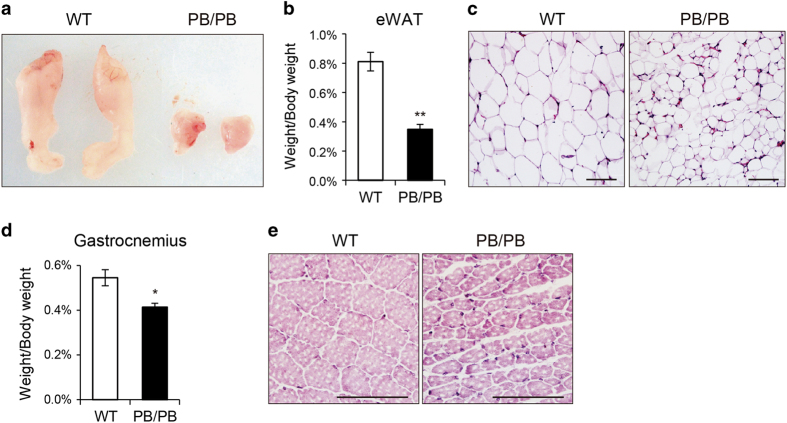
*Samd4*^*PB/PB*^ mice showed impaired adipose and muscle phenotype. (**a**) Representative photographs of epididymal white adipose tissue (eWAT) from 3-week-old male wild-type (WT) and *Samd4*^*PB/PB*^ mice. (**b**) Weights of eWAT normalized to body weight in 3-week-old male WT and *Samd4*^*PB/PB*^ mice. (**c**) Hematoxylin–eosin staining sections of eWAT from 3-week-old WT and *Samd4*^*PB/PB*^mice. (**d**) Weights of gastrocnemius normalized to body weight in 3-week-old male WT and *Samd4*^*PB/PB*^ mice. (**e**) Hematoxylin–eosin staining sections of gastrocnemius from 3-week-old WT and *Samd4*^*PB/PB*^mice. For panels (**b** and **d**), values represent mean±s.d. (*n*=5 for each genotype). *P*-values were obtained from *t*-tests with paired or unpaired samples, *P<0.05, **P<0.01. Bars=100 μm in panels (**c** and **e**).

Recently, a *Samd4* missense mutant mouse model (*spmd*) was generated from *N*-ethyl-*N*-nitrosourea mutagenesis of C57BL/6J mice [37]. The *spmd* mice displayed leanness and myopathy [37]. Mechanistically, Samd4 may interfere with mTORC1 signaling through its interaction with 14-3-3 proteins, and one amino-acid mutation in the SAMD4 protein (SAMD4^H86P^) was shown to disrupt this interaction [37]. To examine whether there are overlapped phenotypes between *Samd4*^*PB/PB*^ mice and *spmd* mice, we also investigated the epididymal white adipose tissue and gastrocnemius of WT and *Samd4*^*PB/PB*^ littermates at 3-week-old age. Indeed, similar to *spmd* mice, *Samd4*^*PB/PB*^ mice exhibited a significant reduction in epididymal white adipose tissue weight (Figure 2a and b) after normalization to total body weight. Histological analysis indicated reduced cell size in white adipose tissue from 3-week-old *Samd4*^*PB/PB*^ mice compared with WT littermates (Figure 2c). In addition, *Samd4*^*PB/PB*^ mice displayed a significant decrease in gastrocnemius weight (Figure 2d), along with abnormalities in the morphology of myofibers (Figure 2e). Collectively, these data indicate that *Samd4*^*PB/PB*^ mice recapitulated the phenotypes of *spmd* mice.

### Samd4^PB/PB^ mice exhibit impaired skeletogenesis

**Figure 3:**
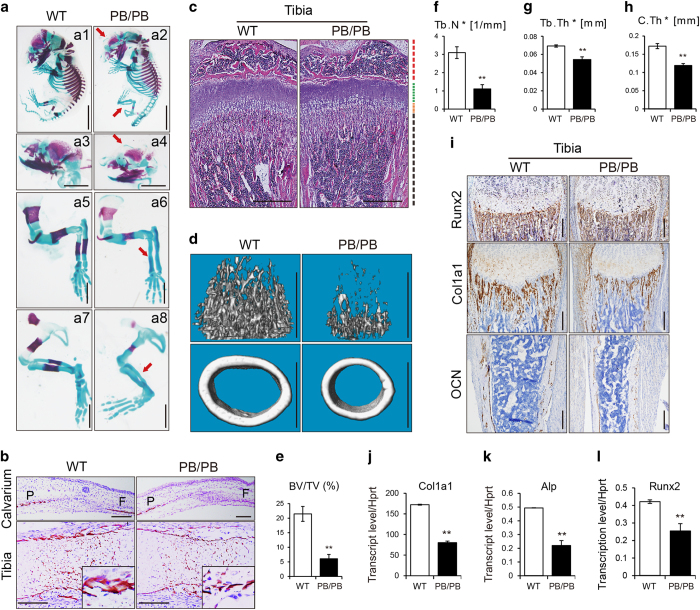
*Samd4*^*PB/PB*^ mice displayed osteopenic phenotype. (**a**) Alizarin red and alcian blue staining of newborn wild-type (WT) and *Samd4*^*PB/PB*^ mice. **a1** and **a2**, whole mount mice; **a3** and **a4**, skulls; **a5** and **a6**, forelimbs; **a7** and **a8**, hindlimbs. Red arrows indicated the lack of alizarin red staining in *Samd4*^*PB/PB*^ mice. (**b**) Von Kossa staining of calvarium and tibia from E16.5 WT and *Samd4*^*PB/PB*^ embryos, counterstained with Nuclear Fast Red. Mineral is stained black; nuclei are stained red. P, parietal bone; F, frontal bone. (**c**) Hematoxylin–eosin staining of tibia of 3-week-old WT and *Samd4*^*PB/PB*^male mice, showing the secondary ossification center (red), the proliferation zone (green), the hypertrophic zone (orange) and the trabecular region (black). (**d**–**h**) Micro-computed tomography analysis of distal femurs from 3-week-old *Samd4*^*PB/PB*^ male mice and WT control mice. (**d**) Representative three-dimensional reconstructions of distal femur trabecular bone. (**e**) The quantitative parameters bone volume/tissue volume (BV/TV), (**f**) trabecular bone number (Tb.N), (**g**) trabecular bone thickness (Tb.Th) and (**h**) cortical bone thickness (C.Th). (**i**) Immunohistochemistry staining of the indicated genes on the sections of tibia from 1-week-old *Samd4*^*PB/PB*^ mice and control WT mice. (**j**–**l**) Analysis of the transcript levels of the indicated genes by quantitative PCR using RNA isolated from 7-day-old WT and *Samd4*^*PB/PB*^ femurs. Values represent mean±s.d. (*n*=6 (**e**–**h**) and 3 (**j**–**l**) for each genotype). *P*-values were obtained from *t*-tests with paired or unpaired samples, **P<0.01. Bars=1 mm in panels (**a** and **d**); 200 μm in panels (**b** and **i**); 500 μm in (**c**).

It is known that myocytes, adipocytes and osteoblasts are differentiated from mesenchymal stem cells. However, *spmd* mice exhibited normal bone mineral density or bone mineral content [37]. On the basis of the wide expression of Samd4 and the reduced skeleton size of *Samd4*^*PB/PB*^ mice, we hypothesized that SAMD4 has effects on skeleton development. We characterized the whole-mount skeleton of mice by alizarin red and alcian blue staining at postnatal day 1. Compared with WT mice, *Samd4*^*PB/PB*^ mice exhibited abnormal skull shape and less alizarin-red-stained craniofacial region, along with delayed ossified long bones (Figure 3a). Bone ossification and mineralization were also examined by von Kossa staining of mice at embryo day 16.5. As shown in Figure 3b, *Samd4*^*PB/PB*^ mice displayed less mineralization compared with the WT mice in both calvarium and tibia. At 3-week-old age, *Samd4*^*PB/PB*^ mice exhibited less trabecula bones compared with WT controls (Figure 3c). To further determine the *in vivo* effects of Samd4 in the skeleton system, we used microquantitative computed tomography to determine the bone density of 3-week-old mice. As shown in Figure 3d–g, *Samd4*^*PB/PB*^ mice exhibited a nearly 75% reduction in femoral trabecular bone volume/total volume (Figure 3d and e), along with a dramatic decrease both in trabecular number (Figure 3d and f) and trabecular thickness (Figure 3d and g), indicating a severe osteopenic phenotype in *Samd4*^*PB/PB*^ mice. In the meantime, cortical thickness was decreased in *Samd4*^*PB/PB*^ mice compared with WT controls (Figure 3d and h). Consistent with the deficiency in osteogenesis and skeletal mineralization, the expression of characteristic osteoblast marker genes, collagen type I alpha 1 (Col1a1), osteocalcin and runt-related transcription factor 2 (Runx2), was reduced in 7-day-old *Samd4*^*PB/PB*^ tibia as determined by immunostaining (Figure 3i). Quantitative reverse transcriptase-PCR (RT-PCR) analyses also confirmed that the expression of osteoblast marker genes, including Col1a1, alkaline phosphatase (Alp) and Runx2, was significantly decreased in the femurs from *Samd4*^*PB/PB*^ mice (Figure 3j–l). Taken together, these data suggested that deficiency of *Samd4* gene impaired bone ossification and mineralization in mice.

### SAMD4 promotes osteoblast differentiation in vitro

**Figure 4:**
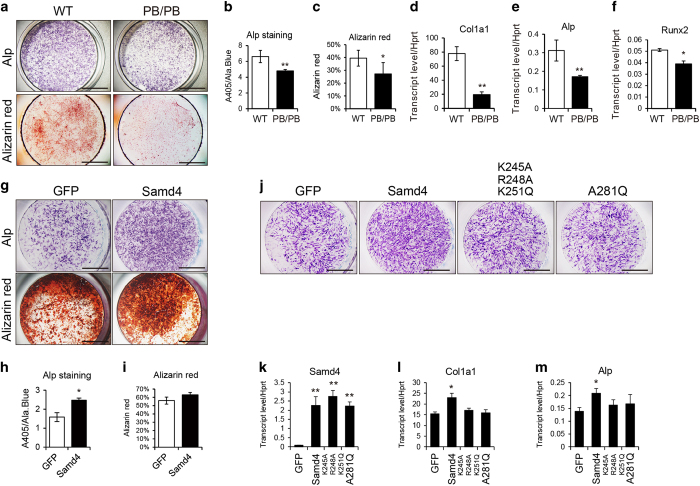
Samd4 (sterile alpha motif domain containing protein 4) affected osteoblastogenesis *in vitro*. (**a**) Representative photographs of fast blue staining (top, cultured in OBD for 7 days) and alizarin red staining (bottom, cultured in OBD for 14 days) of differentiated osteoblasts wild-type (WT) and *Samd4*^*PB/PB*^ mice. (**b**, **c**) Quantitative parameters of Alp activity (**b**) and alizarin red staining (**c**) analyzed by colorimetric assay. (**d**–**f**) Analysis of the transcript levels of the indicated genes by quantitative PCR using RNA isolated from primary osteoblasts of WT and *Samd4*^*PB/PB*^ mice. (**g**) The representative photographs of fast blue staining and alizarin red staining of primary osteoblasts infected with green fluorescent protein (GFP)- and Samd4-expressing lentivirus. (**h**, **i**) Quantitative parameters of Alp activity staining (**h**) and alizarin red staining (**i**) analyzed by colorimetric assay. (**j**) The representative photographs of fast blue staining of osteoblasts differentiated from *Samd4*^*PB/PB*^ osteoblasts infected with lentivrus-expressing GFP, Samd4 or two Samd4 mutants. (**k**–**m**) Analysis of the transcript levels of the indicated genes by quantitative PCR. Values represent mean±s.d. (*n*=6 (**b**, **c**, **h** and **i**) and 3 (**d**–**f** and **k**–**m**) for each genotype). *P*-values were obtained from *t*-tests with paired or unpaired samples, *P<0.05, **P<0.01. Bars=2 mm in panels (**a**, **g** and **j**).

We next examined the effects of Samd4 on osteoblasts differentiation *in vitro*. Primary osteoblasts were collected from calvaria of 7-day-old *Samd4*^*PB/PB*^ and WT mice. After 7 and 14 days of culture under osteoblast differentiation medium, Alp activity was monitored as an early osteoblast differentiation maker and alizarin red staining was monitored as mineral deposition, respectively. Consistent with the osteopenic phenotype in *Samd4*^*PB/PB*^ mice, osteoblasts from *Samd4*^*PB/PB*^ mice revealed an approximately 20% reduction in Alp activity and a significant decrease in alizarin red staining compared with the cells from WT mice (Figure 4a–c), implying that the lack of Samd4 impaired both early and late osteoblast differentiation. Similarly, osteoblasts from *Samd4*^*PB/PB*^ mice revealed decreased the expression of osteoblast markers, including Col1a1, Alp and Runx2 (Figure 4d–f). We next examined the effects of exogenous expression of Samd4. As shown in Figure 4g–i, exogenous SAMD4 could promote osteoblast differentiation determined by Alp activity and alizarin red staining. Taken together, these data indicate that SAMD4 regulates osteoblast differentiation and function *in vitro*.

Considering that *Samd4*^*PB/PB*^ mice displayed significant bone development impairment and Samd4 H86P missense mutant, *spmd*, which has one amino-acid mutation and the interaction with 14-3-3 protein is disrupted, has normal bone density, we hypothesized that RNA-binding activity of SAMD4 is important for skeleton development. SAM is RNA-binding motif in SAMD4. As shown in *Drosophila* SMAUG and *S. cerevisiae* VTS1, several amino acids were crucial for SRE motif binding [38, 39]. To examine whether RNA-binding function of SAM motif is necessary for SAMD4’s effects on bone development, we generated two Samd4 mutants, SAMD4^K245A R248A K251Q^ and SAMD4^A281Q^ (sequences in Supplementary Table S1). We isolated osteoblast from *Samd4*^*PB/PB*^ calvaria, and infected cells with lentivirus-expressing green fluorescent protein, SAMD4 or two SAMD4 mutants, respectively. As shown in Figure 4j–m, SAMD4 could rescue the deficiency of Samd4 in *Samd4*^*PB/PB*^ osteoblasts, while both SAMD4^K245A R248A K251Q^ and SAMD4^A281Q^ were unable to increase osteoblast differentiation determined by Alp staining and the expression of osteoblast marker genes, including Col1a1 and Alp, confirming the requirement of RNA-binding function of SAMD4 in osteoblastogenesis.

### SAMD4 regulates osteoblast differentiation by repressing Mig6 translation

**Figure 5:**
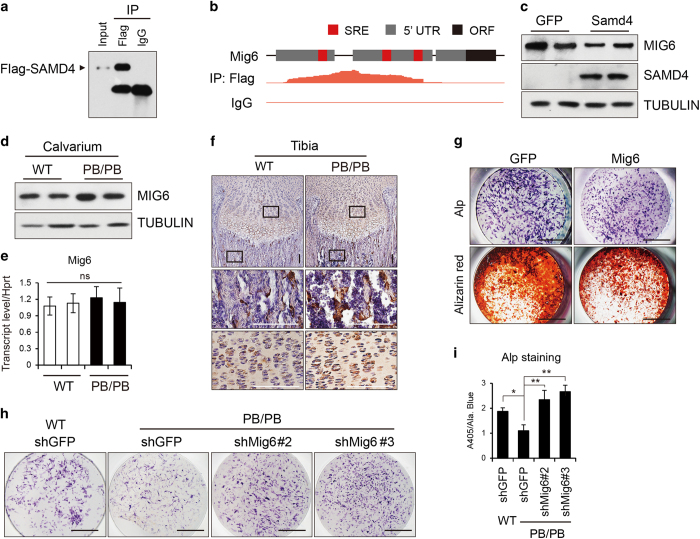
Samd4 (sterile alpha motif domain containing protein 4) bound to mitogen-inducible gene 6 (Mig6) mRNA. (**a**) Western blotting analysis of RNA immunoprecipitation (IP) product reveals Samd4 was enriched in Flag IP. (**b**) Top, DNA structure of 5′ untranslated region (UTR) of Mig6. Bottom, density plot showing the distribution of Flag IP and immunoglobulin G (IgG) peaks according to this region. (**c**) The protein levels of SAMD4 and MIG6 in primary osteoblastswere assessed by western blotting after cells were infected with GFP- or Samd4-expressing lentivirus. (**d**, **e**) The protein (**d**) and mRNA level (**e**) of Mig6 in calvarium from 6-day-old wild-type (WT) and *Samd4*^*PB/PB*^ mice were assessed by western blotting and quantitative PCR, respectively. (**f**) Immunohistochemistry analysis of MIG6 expression on proximal tibia from 1-week-old *Samd4*^*PB/PB*^ mice and control WT mice. (**g**) The representative photographs of fast blue staining and alizarin red staining of osteoblasts infected by GFP- or MIG6-expressing lentivirus. (**h**) The representative photographs of fast blue staining of osteoblasts differentiated from WT and *Samd4*^*PB/PB*^ osteoblasts infected with shRNA lentivirus targeting GFP or Mig6, cultured in OBD for 7 days. (**i**) Quantitative parameters of Alp activity analyzed by colorimetric assay. Values represent mean±s.d. (*n*=3 in panel (**e**) and 6 in panel (**i**) for each genotype). *P*-values were obtained from *t*-tests with paired or unpaired samples, *P<0.05, **P<0.01. Bars=100 μm in panel (**f**) and 2 mm in panels (**g** and **h**).

The dependence of RNA-binding motif SAM domain in SAMD4 to promote osteoblast differentiation suggests that SAMD4 could probably regulate some mRNA targets during osteoblast differentiation through binding to some specific mRNA targets. We next sought to investigate the target mRNAs of Samd4 to investigate its role in translational regulation. An RNA ImmunoPrecipitation (RIP) assay followed by sequencing was performed in osteoblast cells expressing Flag-SAMD4 owing to the lack of a high-quality anti-SAMD4 antibody (Figure 5a). RNA libraries were generated from the RNAs extracted from anti-IgG and anti-Flag samples by using the TruSeq RNA Library Preparation Kit (Illumina). RNA sequencing analysis revealed that 33 genes were enriched in anti-Flag samples compared with anti-IgG samples (Supplementary Table S2). Among those targets, we found that two genes, Mig6 [35, 36] and Tnfrsf12a [40, 41], had been reported to be involved in the regulation of bone development (Figure 5b and Supplementary Table S2).

MIG6 is a non-kinase scaffolding adaptor [33, 34] that is highly expressed in the articular cartilage and growth plates, which comprise both chondrocytes and osteoblasts [35]. Given that Samd4-deficient mice exhibited osteopenic phenotype and that Mig6-deficient mice displayed a subchondral bone phenotype [35, 36], we hypothesized that SAMD4 may regulate Mig6 by binding and inhibiting the translation of Mig6 mRNA. Indeed, upregulating the expression of SAMD4 resulted in a decrease in MIG6 protein level (Figure 5c). We also examined the RNA and protein level of MIG6 in *Samd4*^*PB/PB*^ and WT calvaria from 5-day-old littermates and found increased MIG6 protein level in *Samd4*^*PB/PB*^ calvarium lysates (Figure 5d), whereas there was no obvious difference in RNA level between WT and *Samd4*^*PB/PB*^ mice (Figure 5e). Consistently, immunohistochemical staining analysis revealed more MIG6 protein in 7-day-old *Samd4*^*PB/PB*^ tibia, both in the proliferation zone and trabecular bone zone compared with the WT littermates (Figure 5f). These data support the idea that SAMD4 regulates the translation of *Mig6 in vivo*. However, we could not detect obvious difference of TNFRSF12a protein level between *Samd4*^*PB/PB*^ and WT calvarium lysates (Supplementary Figure S1a and b). Consistent with this, excess SAMD4 could not restrain the protein level of TNFRSF12a (Supplementary Figure S1c). We next determined the effects of increased MIG6 protein and found that overexpression of MIG6 could decrease the differentiation of osteoblasts, as determined by Alp activity and alizarin red staining (Figure 5g).

To further determine whether the decreased osteoblatogenesis in Samd4-deficient cells was due to the dysregulation of MIG6 protein level, we constructed a series of shRNAs (short hairpin RNA) targeting Mig6 (shRNA sequences are shown in Supplementary Table S1). As shown in Supplementary Figure S2, two Mig6-specific shRNAs decreased the transcription of *Mig6*, determined by quantitative RT-PCR. These two shRNAs rescued the decreased osteoblast differentiation in *Samd4*^*PB/PB*^ osteoblasts, compared with WT osteoblasts (Figure 5h and i). We also examined the effects of Mig6 shRNAs on the expression of osteoblast marker genes and confirmed that knockdown of Mig6 could increase the expression of Col1a1, Alp and Runx2 in *Samd4*^*PB/PB*^ osteoblasts (Supplementary Figure S2d–g). These data support that lower osteoblastogenesis of *Samd4*^*PB/PB*^ mice was derived, at least in part, from higher MIG6 protein level.

### SAMD4 binds to SREs on Mig6 mRNA

**Figure 6:**
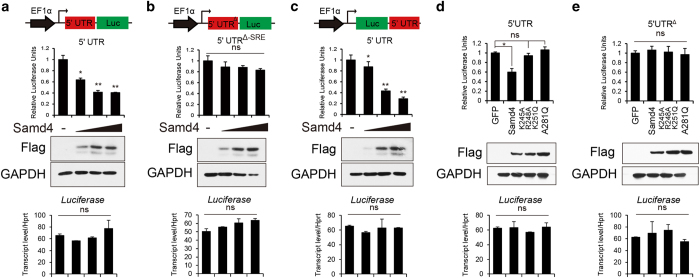
Samd4 (sterile alpha motif domain containing protein 4) bound to 5′ untranslated region (UTR) of mitogen-inducible gene 6 (Mig6) and inhibited MIG6 protein translation in C3H10T1/2. For all panels (**a**–**e**), top, the effects of Samd4 and Samd4 mutants (K245A R248A K251Q and A281Q) were assessed by analyzing the luciferase activity when luciferase cDNA was fused to the indicated elements. Medium, protein level of Flag-Samd4 and glyceraldehyde-3-phosphate dehydrogenase (GAPDH) as the control measured by western blottings. Bottom, relative expression level of luciferase RNA measured by quantitative reverse transcriptase-PCR. (**a**–**c**) Analyses of gradient Samd4 impacted activities of luciferases indicated. Gradient concentrations of SAMD4 were obtained by 0, 50, 200 and 800 ng plasmid transfections, respectively. (**d**, **e**) The effects of Samd4 and Samd4 mutants were assessed by analyzing the luciferase activity. EF1α, EF1α promoter; 5′UTR, 5′UTR of Mig6 mRNA. Values represent mean±s.d. (*n*=3). *P*-values were obtained from *t*-tests with paired or unpaired samples. ns, not significant..

Previous *in vitro* studies showed that mammalian SAMD4 repressed the translation of luciferase carrying SRE motifs [32]. Interestingly, we identified three putative SRE elements at 5′ untranslated region (UTR) of Mig6 mRNA, but not at the 3′UTR. We next examined whether SAMD4 had the effects on the reported SRE sequence and whether the SRE sequence could be located at either 3′UTR or 5′UTR. We performed luciferase reporter assays in C3H10T1/2 cells and found that SAMD4 remarkably downregulated luciferase activity when 3× SRE DNA sequences was fused to the 3′ terminal of luciferase cDNA; in contrast, Samd4 had no effects when those SREs were replaced with the poly A sequence (Supplementary Figure S3a). Strikingly, SAMD4 could also decrease the luciferase activity when 3×SRE sequence was fused to the 5′ terminal of luciferase cDNA (Supplementary Figure S3b), indicating that the effects of SAMD4 on SREs were not restricted to 3′UTR. We next examined the effect of SAMD4 on 5′UTR of Mig6 mRNA. As shown in Figure 6a, SAMD4 could dose-dependently repress luciferase activity when 5′UTR of Mig6 was fused to the 5′ terminal of luciferase cDNA (5′UTR, sequence shown in Supplementary Table S1). Moreover, the effects of the SAMD4 were abolished when the SREs in Mig6 5′UTR were mutated (5′UTR^Δ^, sequence shown in Supplementary Table S1) (Figure 6b). Moreover, SAMD4 could also inhibit luciferase activity in a similar way when the 5′UTR of Mig6 was moved to 3′ end of luciferase cDNA (Figure 6c). We next asked whether RNA-binding function is necessary for SAMD4’s effects on 5′UTR of Mig6 mRNA. As shown in Figures 6d and e, the mutations in the RNA-binding function (SAMD4^K245A R248A K251Q^ and SAMD4^A281Q^) impaired the SAMD4’s effects in this luciferase activity assay. These data suggest that SAMD4 could bind to 5′UTR of Mig6 mRNA and repress MIG6 protein translation.

### SAMD4 promotes chondrogenesis and chondrocyte differentiation

**Figure 7:**
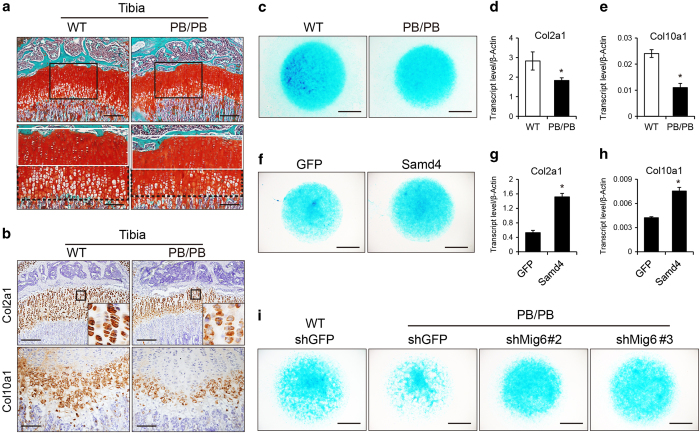
Samd4 (sterile alpha motif domain containing protein 4) influenced chondrogenesis through mitogen-inducible gene 6 (Mig6). (**a**) Safranin O staining of tibia from 3-week-old wild-type (WT) and *Samd4*^*PB/PB*^male mice, showing the proliferation zone (white box) and the hypertrophic zone (black box). (**b**) Immunohistochemical staining of the indicated genes on the sections of tibia from 3-week-old *Samd4*^*PB/PB*^ mice and control WT mice. (**c**) Representative photographs of alcian blue staining of chondrocytes separated from WT and *Samd4*^*PB/PB*^ articular cartilage. (**d**, **e**) Analysis of the transcript levels of genes by quantitative PCR using RNA isolated from chondrocytes of WT and *Samd4*^*PB/PB*^. (**f** and **i**) Representative photographs of alcian blue staining of chondrocytes differentiated from WT and *Samd4*^*PB/PB*^ articular cartilage and infected with lentivrus indicated, respectively. (**g** and **h**) Analysis of the transcript levels of genes by quantitative PCR using RNA isolated from chondrocytes of WT and *Samd4*^*PB/PB*^ that had been infected with the lentivirus-expressing enhanced GFP or Samd4. β-Actin, beta-actin. Values represent mean±s.d. (*n*=3 in panels (**d**, **e**, **g** and **h**) for each genotype). *P*-values were obtained from *t*-tests with paired or unpaired samples, *P<0.05. Bars=100 μm in panels (**a** and **b**) and 1 mm in panels (**c**, **f** and **i**).

Cartilage-specific deletion of Mig6 was shown to result in osteoarthritis-like disorder with excessive articular chondrocyte [35, 36]. The repression of MIG6 expression by SAMD4 implied that SAMD4 have effects on chondrocyte. *Samd4*^*PB/PB*^ mice dwarfed remarkably in contrast to WT littermates (Figure 1f), supporting the chondrocyte phenotype in *Samd4*^*PB/PB*^ mice. We examined the growth plate of 3-week-old *Samd4*^*PB/PB*^ mice tibia by safranin O staining. As shown in Figure 7a, *Samd4*^*PB/PB*^ mice displayed shortened resting and columnar zones, although the growth plate was almost normal-organized. Consistently, the expression of characteristic chondrocyte marker genes, collagen type II alpha 1 (Col2a1) and collagen type X alpha 1 (Col10a1) was reduced in 3-week-old *Samd4*^*PB/PB*^ tibia as determined by immunostaining (Figure 7b). We also did chondrocyte micromass culture and found that chondrocytes from *Samd4*^*PB/PB*^ mice revealed significant reduction in alcian blue staining (Figure 7c). Consistently, chondrocytes cultured from *Samd4*^*PB/PB*^ mice revealed decreased expression of marker genes, including Col2a1 and Col10a1 (Figure 7d and e). We also examined the effects of exogenous expression of SAMD4 in chondrocytes and found that SAMD4 could promote chondrocyte differentiation (Figure 7f–h). Next we explored the dependence of Mig6 for the augmented chondrocyte differentiation in *Samd4*^*PB/PB*^cells. As shown in Figure 7i and Supplementary Figure S4a–c, two Mig6-specific shRNAs increased chondrocyte differentiation determined by alcian blue staining and the expression levels of marker genes in chondrocytes from *Samd4*^*PB/PB*^ mice. Taken together, these data indicate that SAMD4 regulate chondrogenesis and chondrocyte differentiation and function *in vivo* and *in vitro*, consistent with the mechanism by which deficiency of Samd4 in osteoblast results in higher MIG6 protein level.

## Discussion

Our current study demonstrates that RNA-binding protein SAMD4 could regulate osteoblast and chondrocyte differentiation, indicating that SAMD4 is a novel regulator for skeletogenesis. *Samd4*^*PB/PB*^ mice exhibit multiple bone defects, including shortened body length, decreased bone mass, hypomineralization and so on (Figures 1f and g and 3a–h). Furthermore, SAMD4 promotes osteoblast and chondrocyte differentiation *in vitro* (Figures 4a–i and 7c and d). Previous reports showed that the *spmd* mice have normal bone mineral density or bone mineral content [37]. The difference of skeleton phenotype between these two mice models could be explained by the fact that *spmd* mutant only affects a histidine (H86P) in the protein [37]. However, *Samd4*^*PB/PB*^ mice are accompanied with the deletion of whole SAMD4 protein. The requirement of SAM domain for SAMD4 function in osteoblast and chondrocyte supports that the severe skeleton phenotype of *Samd4*^*PB/PB*^ mice is through the depletion of whole protein (Figures 4j–m and 7e–h). Furthermore, we detected that, similar to SAMD4, SAMD4^H86P^ inhibited the activity of luciferase constructs containing SREs (Supplementary Figure S5a and b) indicating that the H86P mutation did not disrupt the function of SAMD4 as a translational repressor, which was demonstrated by the result that SAMD4^H86P^ could rescue the impaired differentiation of *Samd4*^*PB/PB*^ osteoblasts (Supplementary Figure S5c).

The decreased bone mass present in the long bones of *Samd4*^*PB/PB*^ mice may also result from increased osteoclast differentiation and/or function. To address this, we analyzed tibia from 3-week-old WT and *Samd4*^*PB/PB*^ mice for the presence of tartrate-resistant acid phosphatase (TRAP)-positive osteoclasts. In comparison with age-matched WT controls, *Samd4*^*PB/PB*^ mice showed similar numbers of TRAP-positive cells in the proximal tibia of 3-week-old mice (Supplementary Figure S6a). Consistently, the expression of characteristic osteoclast marker genes, including cathepsin K (Ctsk), matrix metallopeptidase 9 (Mmp9), osteoclast-associated receptor (Oscar), dendritic cell-specific transmembrane protein (Dc-stamp) and nuclear factor of activated T cells and cytoplasmic 1 (Nfatc1), was not significantly different between *Samd4*^*PB/PB*^ mice and WT bone marrow (Supplementary Figure S6b). Further supporting these *in vivo* results, culture of bone marrow harvested from *Samd4*^*PB/PB*^ mice and WT mice in the presence of macrophage colony-stimulating factor and receptor activator of NF-κB ligand resulted in the formation of a similar number of multinucleated TRAP-positive osteoclasts (Supplementary Figure S6c). Further analysis of these cultures via quantitative PCR (qPCR) revealed that the osteoclast-specific markers, including Ctsk, Mmp9, Oscar, Dc-stamp and Nfatc1, were induced to similar levels in *Samd4*^*PB/PB*^ mice and WT cultures (Supplementary Figure S6d). Collectively, these results suggest that the osteopenic phenotype observed in *Samd4*^*PB/PB*^ mice is due to decreased bone formation by osteoblasts as opposed to augmented bone resorption by osteoclasts.

A list of SAMD4 target mRNAs are identified by RIP assay, among which, Mig6 is enriched at the top of 33 genes (Figure 5b and Supplementary Table S2). Our study showed that SAMD4 impaired MIG6 protein level, and the deficiency of SAMD4 in *Samd4*^*PB/PB*^ results in higher level of MIG6 protein (Figure 5d). Knockdown of Mig6 rescues the decreased osteoblast differentiation in Samd4-deficient osteoblasts, supporting that higher protein level of MIG6 in Samd4-deficient osteoblasts is responsible for the impaired osteoblastogenesis (Figure 5h and i and Supplementary Figure S2). Furthermore, we discover that SAMD4 could block the translation of luciferase when 5′UTR of Mig6 mRNA is located in 5′ or 3′ end of luciferase mRNA (Figure 6a–c). Mig6 has been demonstrated as having an essential role in chondrocyte proliferation and differentiation [35, 36]. The chondrocyte defects in *Samd4*^*PB/PB*^ mice (Figure 7 and Supplementary Figure S4) are consistent with our description of the mechanism by which SAMD4 represses the Mig6 protein translation. Thus the decreased bone density of *Samd4*^*PB/PB*^ mice could be related to the protein translational regulation by SAMD4.

Overall, this study demonstrates that mammalian Samd4 is a translational repressor that inhibits Mig6 expression and regulates skeletal development. However, the function of the regulation of protein synthesis in skeletal development remains elusive. In this study, we identified mammalian SAMD4 as a novel regulator of osteogenesis, and our results suggest that control of protein translation could have a therapeutic potential in metabolic bone diseases, such as osteoporosis.

## Materials and Methods

### Mouse lines

*Samd4*^*PB/PB*^ mice were obtained from the PB Mutagenesis Center, Institute of Developmental Biology and Molecular Medicine, Fudan University. All mice analyzed were maintained on an FVB background. All animals were maintained in pathogen-free conditions in accordance with the guidelines of the Institute of Biochemistry and Cell Biology, Shanghai Institutes for Biological Sciences, Chinese Academy of Sciences, Shanghai, China.

### Cell culture

Human embryonic kidney cell line (HEK293T) was obtained from American Type Culture Collection (ATCC, Manassas, VA, USA) and maintained in high glucose minimum essential medium (Dulbecco’s modified Eagle’s medium) containing 10% fetal bovine serum (FBS). Mouse embryonic fibroblast cells (C3H10T1/2) were obtained from ATCC and maintained in minimum essential medium (MEM) alpha containing 10% FBS. Primary murine osteoblasts were isolated from calvaria of P5–P7 mice as described previously [42]. For osteoblast differentiation, primary osteoblasts were cultured in low glucose alpha MEM containing 10% FBS and induced in 50 μg ml^−1^ L-ascorbic acid and 1080 mg ml^−1^ β-glycerophosphate. The osteoblast differentiation level assay was performed following a previously published method [43]. The isolation and differentiation of primary osteoclasts were also previously described [44]. For the chondrogenic differentiation, the chondrocytes were separated from the articular cartilage from P3 to P5 mice and cultured in low glucose alpha MEM containing 10% FBS. A two-dimensional micromass culture was initiated by spotting 2.5×10^5^ chondrocytes in 15 μl to each well of a 12-well-plate and maintained in chondrogenesis medium supplemented with 500 nM ml^−1^ TGFβ3 (R&D Systems, Lille, France, 243-B3-200). After 7 days of differentiation, the micromass culture was stained by 1% alcian blue.

### Histological analyses

For histological analyses, tissues were fixed in 4% paraformaldehyde overnight at 4 °C. For gastrocnemius and epididymal white adipose tissue, samples were dehydrated in 60% sucrose/phosphate-buffered saline solution overnight at room temperature and then embedded in an optimal cutting temperature compound (Tissue-Tek, Sakura, Torrance, CA, USA) under a stereomicroscope and transferred onto dry ice to solidify. Postnatal tibia samples were decalcified in 20% EDTA for 14 days (P7) and 3-week-old. Decalcified samples were dehydrated using graded ethanol overnight and embedded in paraffin. Paraffin-embedded tissue samples were sectioned into 8 μm sections. Immunohistochemical staining was performed as previously described [45]. Images were captured using a microscope (Olympus BX51, Tokyo, Japan). For TRAP staining, the sections of tibia from 3-week-old littermates were fixed in 100% methanol for 5 min, washed 5 min in phosphate-buffered saline twice and then stained using the Leukocyte Acid Phosphatase (TRAP) Kit (Sigma, 387 A-1KT, St Louis, MO, USA).

### Microquantitative computed tomography analysis

Femurs isolated from age- and sex-matched 3-week-old mice were fixed in 70% ethanol and scanned using SkyScan1176 (Bruker, Kartuizersweg, Belgium). Femurs and skulls were scanned at 8.96-micron resolution. For analysis of femoral bone mass, a region of trabecular bone 2.0 mm wide was contoured, starting 600 microns from the proximal end of the distal femoral growth plate. For femoral trabecular bone, a threshold of 80–255 permille was used. The region of interest of the femoral cortical bone was 1.0 mm wide, starting 3.7 mm from the proximal end of the distal femoral growth plate. For femoral cortical bone, a threshold of 125–255 permille was used. Three-dimensional reconstructions were created by stacking the two-dimensional images from the contoured regions.

### Real-time RT-PCR

Total RNA was prepared from cells or tissues using TRIzol (T9424, Sigma) and reverse transcribed into cDNA using the PrimeScript RT Reagent Kit (PR037A, TakaRa, Dalian, China). The cDNA quantity was analyzed by the BioRad CFX96 system (BioRad, Hercules, CA, USA). The primer sets used were as follows: Hprt sense 5′- GTTAAGCAGTACAGCCCCAAA-3′ and antisense 5′- AGGGCATATCCAACAACAAACTT-3; Samd4 sense 5′- CTCTCGGGATTCTGGGATTTGC-3′ and antisense 5′- TACTTGTGCAGGCGGAGGCTT-3; Alp sense 5′- GGGCAACTCCATCTTTGGTCTG-3′ and antisense 5′- TTCACCGTCCACCACCTTGT-3′; Col1a1 sense 5′- GCTCCTCTTAGGGGCCACT-3′ and antisense 5′- CCACGTCTCACCATTGGGG-3′; Runx2 sense 5′- ATGATGACACTGCCACCTCTGACT-3′ and antisense 5′- ATGAAATGCTTGGGAACTGCCTGG-3′; Tnfrsf12a sense 5′- CAGGACCCGTCGGAACTGAATC-3′ and antisense 5′- CAGCCACTCACTGTCCCGTCCA-3′; Mig6 sense 5′- GCCTACAATCTGAACTCCCCTGC-3′ and antisense 5′- TAATGGGTGTAAGTGGAGGTGTGGA-3′; Col2a1 sense 5′- CGGTCCTACGGTGTCAGG-3′ and antisense 5′- GCAGAGGACATTCCCAGTGT-3′; Col10a1 sense 5′- CCCACAGGCATAAAGGGCCCAC-3′ and antisense 5′- GGACCTGGGTGTCCTCGAGGTCC-3′; Ctsk sense 5′- GAAGAAGACTCACCAGAAGCAG-3′ and antisense 5′- TCCAGGTTATGGGCAGAGATT-3; Mmp9 sense 5′- CTGGACAGCCAGACACTAAAG-3′ and antisense 5′- CTCGCGGCAAGTCTTCAGAG-3′; Nfatc1 sense 5′- GACCCGGAGTTCGACTTCG-3′ and antisense 5′- TGACACTAGGGGACACATAACTG-3′; Oscar sense 5′- CCTAGCCTCATACCCCCAG-3′ and antisense 5′- CGTTGATCCCAGGAGTCACAA-3′; and Dc-stamp sense 5′- GGGGACTTATGTGTTTCCACG-3′ and antisense 5′- ACAAAGCAACAGACTCCCAAAT-3′.

### RIP and RNA sequencing

A total of 10^7^ Flag-Samd4-expressing *Samd4*^*PB/PB*^ osteoblasts were resuspended in IP buffer (50 mM Tris-HCl Ph 7.5, 150 mM NaCl, 1% NP-40, 0.5% sodium deoxycholate and 1 mM phenylmethanesulfonyl fluoride), subjected to gentle sonication and centrifuged to obtain cell extracts. Protein–RNA complex immunoprecipitation and RNA sequencing was performed as previously described [46]. The RIP-seq data reported in this study have been deposited in NCBI’s GEO, series accession number GSE90818.

### Luciferase reporter assays

C3H10T1/2 cells plated on 12-well-plates were transiently transfected using polyetherimide (Sigma, 408727) with the luciferase reporter constructs and pRL-TK (Promega, Madison, WI, USA) as a normalization control for transfection efficiency, together with plasmids encoding SAMD4 and SAMD4 mutants. Forty-eight hours after transfection, the cells were lysed and luciferase activity was measured using the Dual Luciferase Assay.

### Antibodies

Anti-RUNX2 antibody (SC-390351), anti-SAMD4 antibody (SC-163329) and anti-TUBULIN antibody (SC-23948) were from Santa Cruz Biotechnology (Santa Cruz, CA, USA); monoclonal anti-Flag antibody (F3165) was from Sigma-Aldrich; anti-MIG6 antibody (11630) was from ProteinTech, Rosemont, IL, USA; and anti-Tnfrsf12a (ab109365) was from Abcam (San Francisco, CA, USA).

### Plasmids and lentivirus

To generate the Flag-Samd4 construct, Samd4 was amplified from the total cDNA library of 10.5-day-old embryonic mice (E10.5) and cloned into the modified pHAGE vector containing Flag using restriction enzymes Not I and BamH I. The full-length Samd4 open reading frame was obtained from total mouse cDNA and inserted into pHAGE vector using restriction enzymes Not I and BamH I. The targeting sequences of specific shRNAs, which are listed in Supplementary Table S1, were cloned into pLKO.1 lentiviral vectors, and the insertions were confirmed by DNA sequencing. The lentivirus were produced by co-transfection of plasmids with VSVG and Δ8.9 plasmids into HEK293T cells following the protocol from Open Biosystems (GE Healthcare).

### Statistical analysis

For statistical analysis, in most graphs mean values with s.d. are shown. Data were generated from several independently replicates. *P*-values were obtained from two-tailed Student’s *t*-tests. *P-*value<0.05 was considered statistically significant.
