# Supplementary material for: RNA-binding protein SAMD4 regulates skeleton development through translational inhibition of Mig6 expression
Source: Cell Discov. 2017 Jan 24;3:16050–. doi: 10.1038/celldisc.2016.50 (PMC5259697; doi:10.1038/celldisc.2016.50)
Supplement: Supplementary Information [file celldisc201650-s1.pdf]

Supplemental Information

RNA binding protein SAMD4 regulates skeleton development through translational inhibition of Mig6 expression

Ningning Niu[1], Jian-Feng Xiang[2], Qin Yang[3], Lijun Wang[1], Zhanying Wei[4], Ling-Ling Chen[2], Li Yang[3], Weiguo Zou[1]

Inventory of supplemental information

Figure S1, related to Figure 5

Figure S2, related to Figure 5

Figure S3, related to Figure 6

Figure S4, related to Figure 7

Figure S5

Figure S6

Table S1, DNA and peptide sequences

Table S2, Genes enriched in Flag IP

Supplementary Information

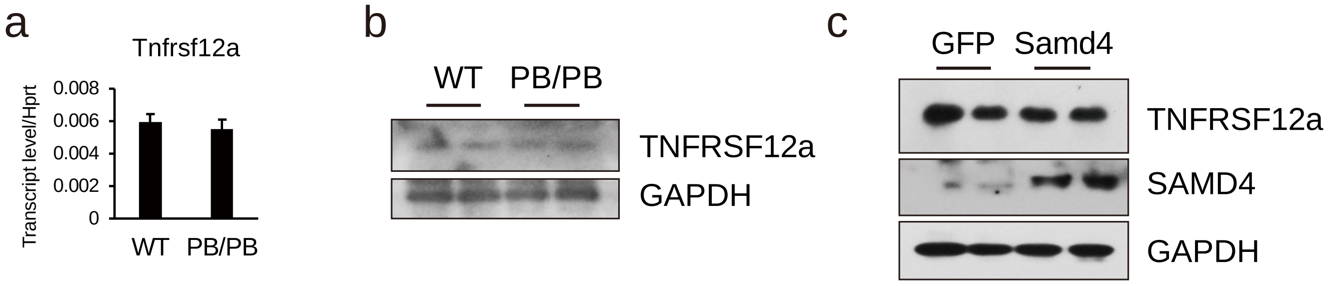

Supplementary Figure 1. Samd4 couldn't restrain the expression of Tnfrsf12a. (**a**, **b**) The mRNA (**a**) and protein level (**b**) of Tnfrsf12a in calvarium from 6-day old WT and *Samd4*$^{PB/PB}$ mice were assessed by quantitative RT-PCR and western blot, respectively. (**c**) The protein levels of SAMD4 and Tnfrsf12a were assessed by western blot after infected with GFP or Samd4–expressing lentivirus.

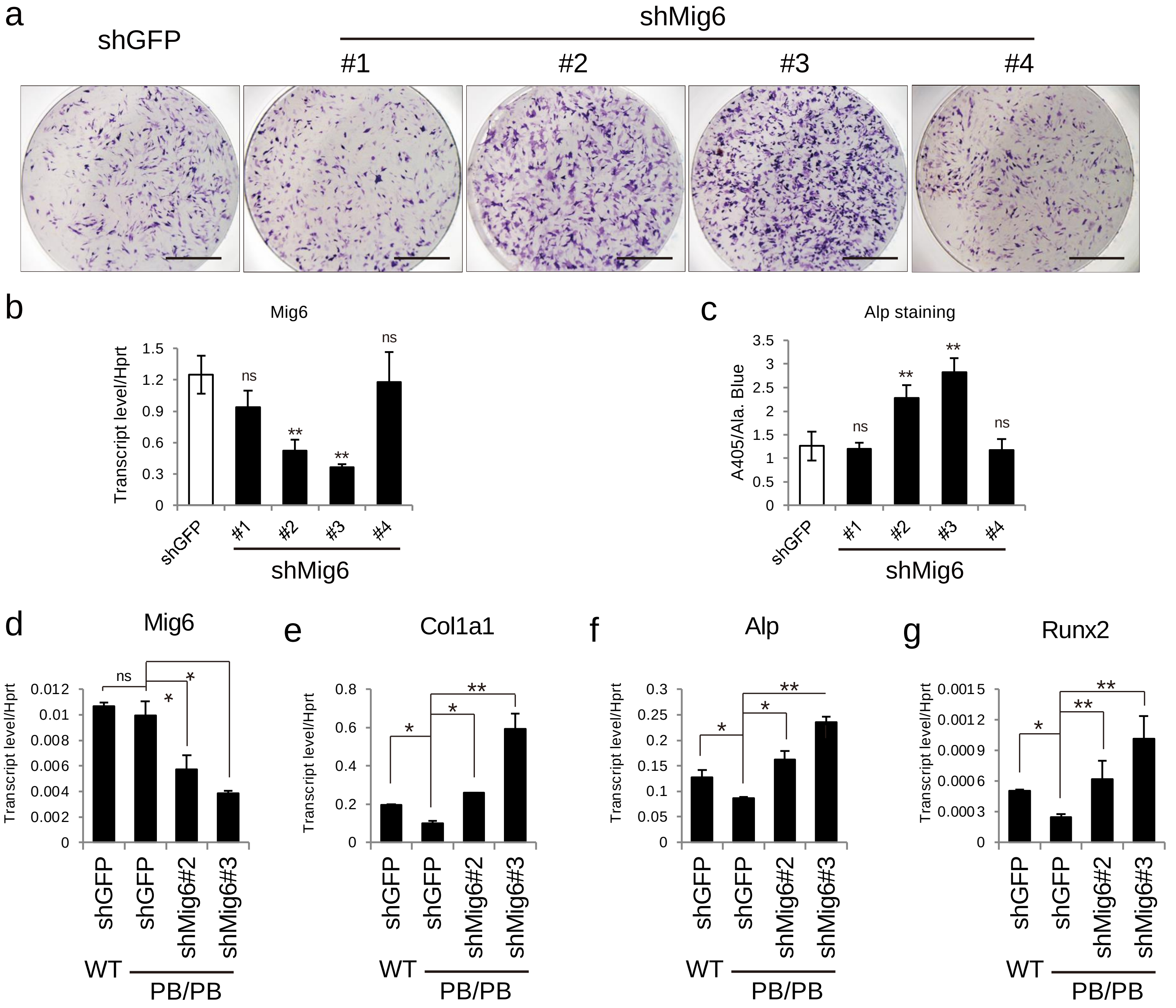

Supplementary Figure 2. Increased osteoblast differentiation in Mig6 knockdown cells. (**a**) Representative photographs of Fast Blue staining of primary osteoblasts infected with shRNA lentivirus targeting GFP or Mig6, cultured in OBD for 7 days. (**b**) Quantitative PCR analysis of Mig6 expression. (**c**) Quantitative parameters of Alp activity analyzed by colorimetric assay. (**d**-**g**) Quantitative PCR analysis was performed for the indicated genes in RNA isolated from WT and *Samd4*$^{PB/PB}$ osteoblasts infected with shRNA lentivirus targeting GFP or Mig6. Values represent mean ± SD (n = 3 (**b, d-g**) and 6 (**c**) for each genotype). *P*-values were obtained from *t*-tests with paired or unpaired samples, ***P* < 0.01. Bars = 2 mm in (**a**).

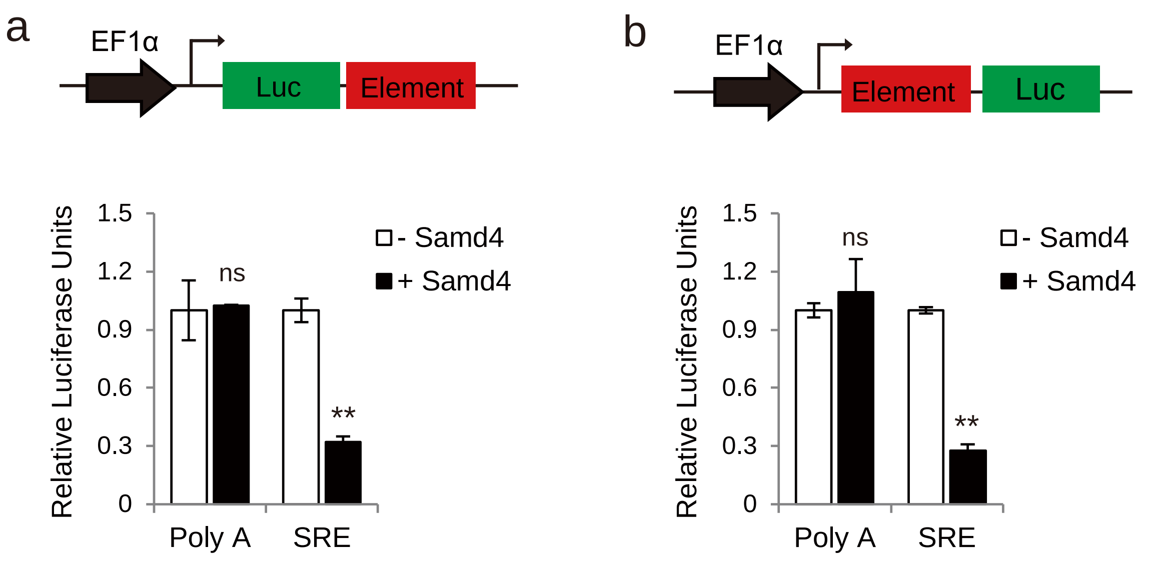

Supplementary Figure 3. Samd4 inhibits SRE element, which could localize at both 5' UTR and 3' UTR, assessed by luciferase activity. (**a**) Samd4 repressed luciferase activity when SRE was inserted on the 3' UTR. (**b**) Samd4 repressed luciferase activity when SRE was inserted on the 5' UTR. Top, luciferase reporter constructs. Bottom, relative luciferase activity. EF1α, EF1α promoter. Values represent mean ± SD (n = 3). *P*-values were obtained from *t*-tests with paired or unpaired samples, ***P* < 0.01.

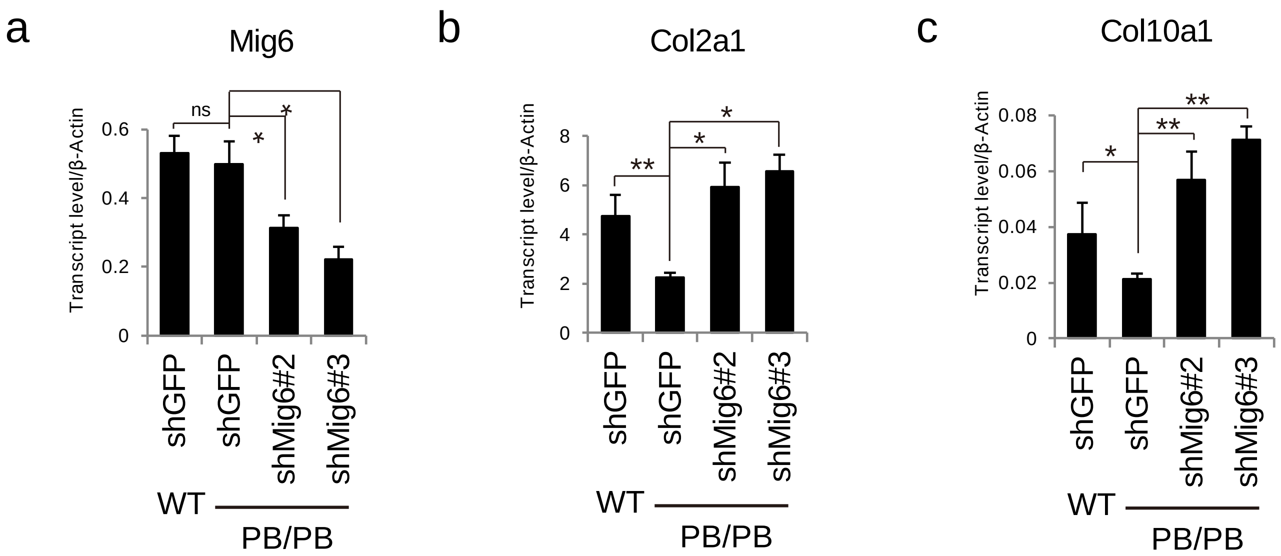

Supplementary Figure 4. The effects of Samd4 and Mig6 on chondrogenesis. (**a**-**c**) Analysis of the transcript levels of genes by quantitative PCR using RNA isolated from chondrocytes of WT and *Samd4*$^{PB/PB}$ which had been infected with the lentivirus expressing the genes as indicated. Values represent mean ± SD (n = 3 for each genotype). *P*-values were obtained from *t*-tests with paired or unpaired samples, *$P < 0.05$.

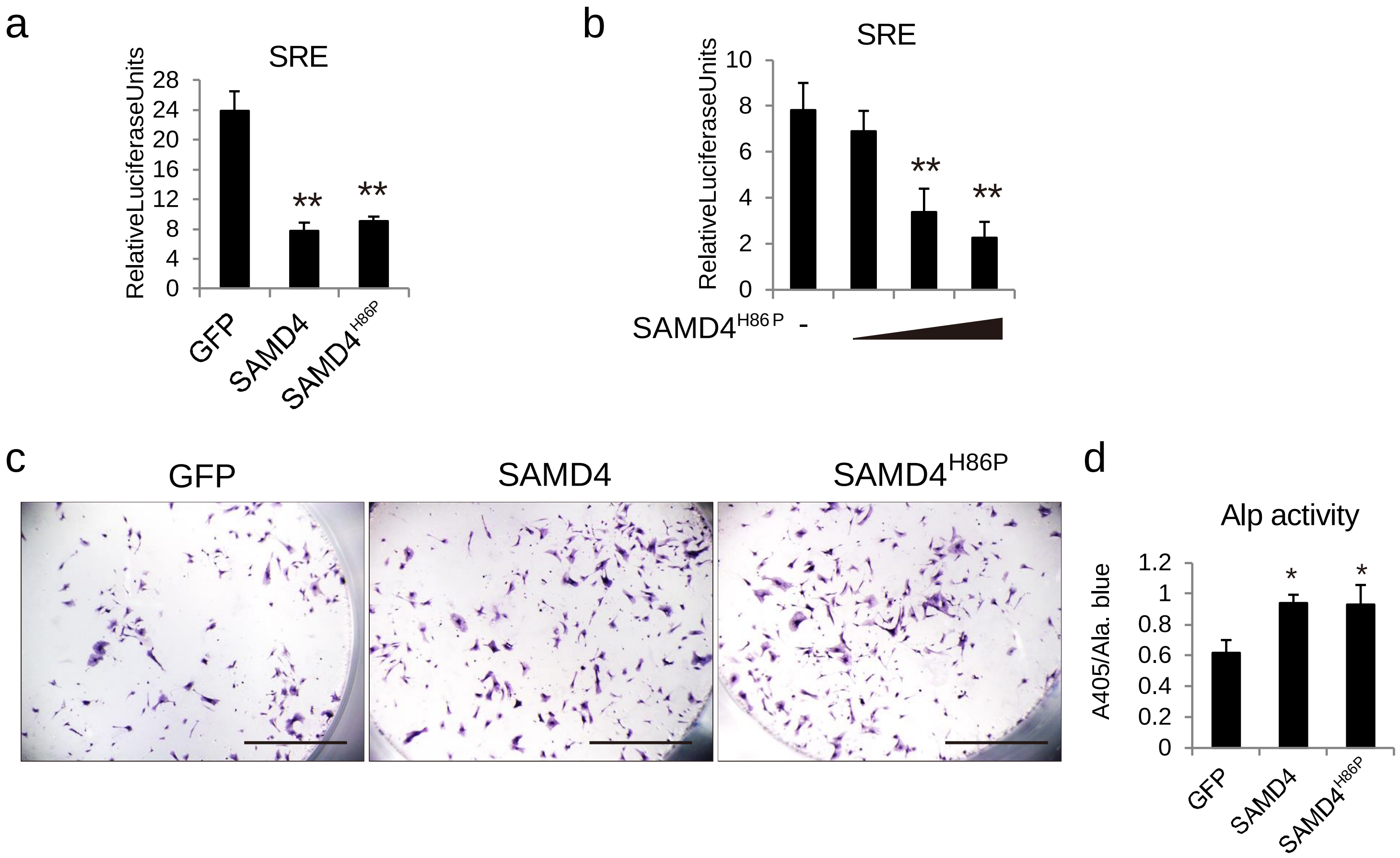

Supplementary Figure 5. SAMD4^{H86P} could not inhibit the activity of luciferase fused to SRE and osteoblastogenesis. (**a**) The effects of SAMD4 and SAMD4^{H86P} were assessed by analyzing the luciferase activity when luciferase cDNA was fused to the indicated elements. (**b**) SAMD4^{H86P} showed gradient inhibition on activities of SRE luciferase. Gradient concentrations of SAMD4^{H86P} were obtained by 0, 50, 200, 800 ng plasmids transfection respectively. (**c**) Representative photographs of fast blue staining and alizarin red staining of primary osteoblasts infected with lentivirus indicated. (**d**) Quantitative parameters of Alp activity analyzed by colorimetric assay. Values represent mean ± SD (n = 3 (**a, b)** and 6 (**d**) for each genotype). *P*-values were obtained from *t*-tests with paired or unpaired samples, *$P < 0.05$, **$P < 0.01$. Bars = 2mm in (**c**).

a

b

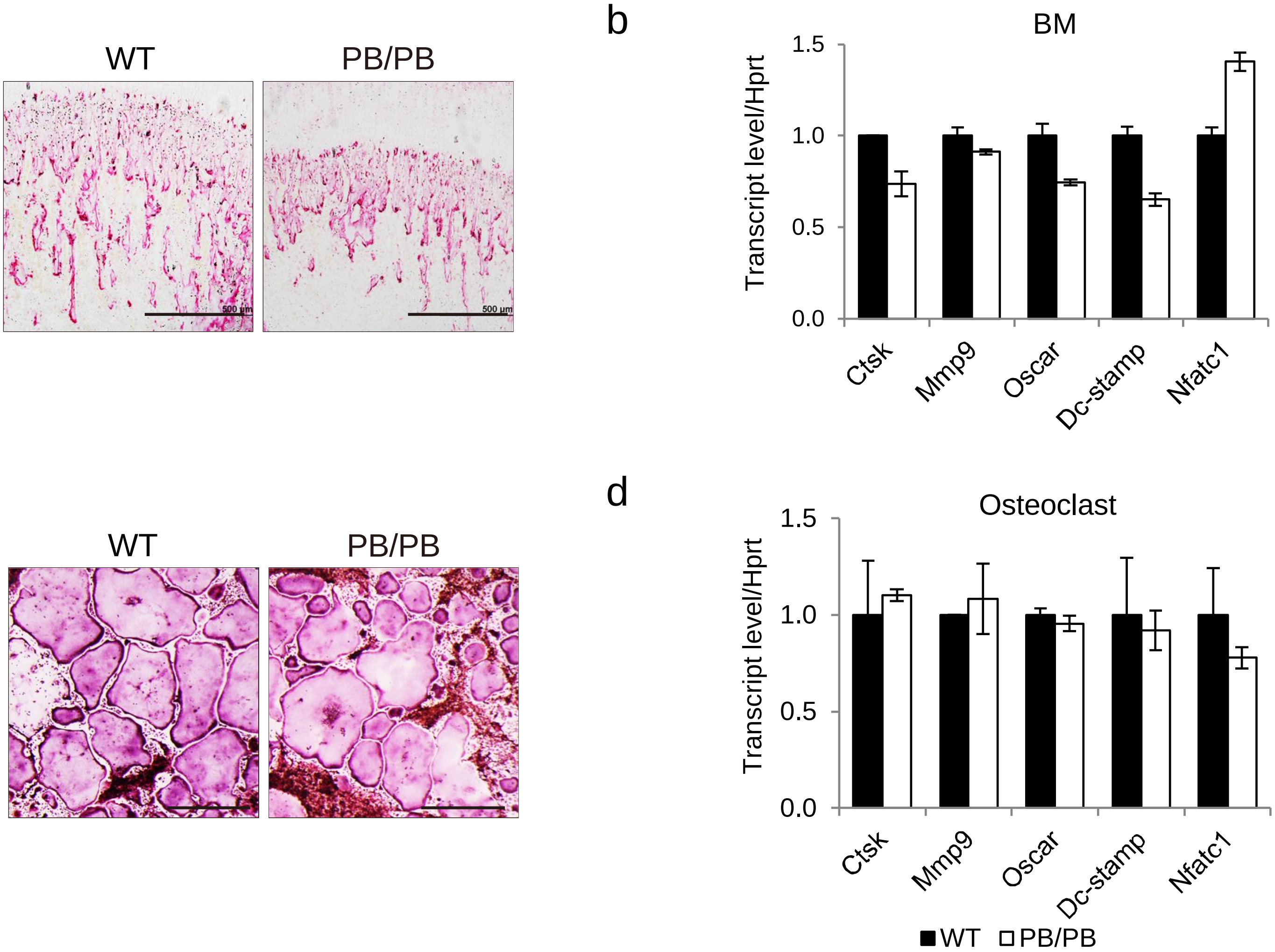

c

d

Supplementary Figure 6. The lack of Samd4 has no obvious effect on osteoclast differentiation. (**a**) TRAP staining (red) in the proximal tibia of 3-week-old WT and *Samd4*$^{PB/PB}$ mice. (**b**) The expression of indicated genes was analyzed by quantitative-PCR analysis in WT and *Samd4*$^{PB/PB}$ bone marrow (BM). (**c**) The representative photographs of TRAP staining of osteoclasts. The bone marrow cells were isolated from WT and *Samd4*$^{PB/PB}$ mice and treated with RANKL for 7 days to undergo osteoclast differentiation. (**d**) The expression of indicated genes was analyzed by quantitative-PCR analysis. Bars = 500 µm in (**a**) and 1 mm in (**c**). Values represent mean ± SD (n = 3).

Supplementary Table S1. DNA and peptide sequences.

	DNA Sequence
**3 x SRE DNA**	GATCTGCAGAGGCTCTGGCAGCTTTTGCCCCGTAAGCGCCTGGCGC<br>GTTTCTAGAAATAATAATCAGCTGGCCTGATTAGTCTAGA
**Poly A DNA**	GACTCTAGATCATAATCAGCCATACCACATTTGTAGAGGTTTTACTTG<br>CTTTAAAAAACCTCCCACACCTCCCCCTGAACCTGAAACATAAAATG<br>AATGCAATTGTTGTTGTTAACTTGTTTATTGCAGCTTATAATGGTTACA<br>AATAAAGCAATAGCATCACAAATTTCACAAATAAAGCATTTTTTTCACT<br>GCATTCTAGT
**5' UTR DNA**	GCGGAGCGTGAGCTGTGCGAGCGAGCGAGCGCGAGCATAGCCTGC<br>GAGCGAGCAGAGAGAAAGAGCGAGGGCAAGAGAGCGGCGAGGCG<br>CCTGCGCGATGCTCGGGCCCCTAAGCCCGCGGCGCTGAGCCAGCC<br>GGGACGGACATGCGCGGGAGGGCGCCGCGGGGTCCCGCTCCCTT<br>GGGGCCTGGGGCTCCGGAGCGGCGGCTCCACGGTCTTCCCGGTGC<br>AGCTGCGGCCCAGGGATGCGGTGGAACTGCTGCGGCCCCCTCCAT<br>CTTCCCTCTCACCCCGCGCACCCGTCGGTCCGGCCCGGAAAGCTTC<br>TGCGAGAAGCTGTCGCGGGTGGGCTCGGA
**5' UTR$^{\Delta}$ DNA**	GCGGAGCGTGAGCTGTGCGAGCGAGCGAGCGCGAGCATAGCCTGC<br>GAGCGAGCAGAGAGAAAGAGCGAGGGCAAGAGAGCGGCGAGGCG<br>CCTGCGCGATGCTCGGGCCCCTAAGCCCGCGGCGCTGAGCCAGCC<br>GGGACGGACATGGCTTTAAAAAACCCGCGGGGTCCCGCTCCCTTGG<br>GCGCTTTAAAAAACCAGCGGCGGCTCCACGGTCTTCCCGGTGCAGC<br>TGCGGCCCAGGGATGCGGTGGAACTGCTGCGGCCCCCTCCATCTT<br>CCCTCTCACCCCGCGCACCCGTCGGTGCTTTAAAAAACGCTTCTGC<br>GAGAAGCTGTCGCGGGTGGGCTCGGA
**shGFP**	GCAAGCTGACCCTGAAGTTCA
**shMig6#1**	GCGTGAAGAGGATCAAGTTAT
**shMig6#2**	CCTTCAGATTTCAAATACGAT
**shMig6#3**	CCACCGTACCTGGACAAATAT

**shMig6#4**	CCTTGCATCCTGCCCATTATT
	**Peptide Sequence**
**SAMD4**	MMFRDQVGVLAGWFKGWNECEQTVALLSLLKRVSQTQARFLQLCL<br>EHSLADCAELHVLEGEANSPGIINQWQQESKDKVISLLLTHLPLLKPG<br>NLDAKAEYMKLLPKILAHSIEHNQHIEESRQLLSYALIHPATSLEDRSA<br>LAMWLNHLEDRTSTSFGSQNRGRSDSVDYGQTHYYHQRQNSDDK<br>LNGWQNSRDSGICISASNWQDKSLGCENGHVPLYSSSSVPATINTIG<br>TGASTNVPAWLKSLRLHKYAALFSQMTYEEMMALTECQLEAQNVTK<br>GARHKIVISIQKLKERQNLLKSLERDIIEGGSLRTPLQELHQMILTPIKA<br>YSSPSTTPEVRCREPSLMESPSPDCKDSAAAVTSATASASAGASGG<br>LQPPQLSSCDGELAVAPLPEGDLPGQFTRVMGKVCTQLLVSRPDEE<br>NISSYLQLLDKCLVHEAFTETQKKRLLSWKQQVQKLFRSFPRKTLLDI<br>SGYRQQRNRGFGQSNSLPTASSVGSGMGRRNPRQYQIASRNVPS<br>ARLGLLGTSGFVSSNQRHTAANPTIMKQGRQNLWFANPGGSNSMP<br>SRTHSSVQKTRSLPVHTSPQNMLMFQQPEFQLPVTEPDINNRLESL<br>CLSMTEHALGDGVDRTSTI-
SAMD4$^{K245A\ R248A\ K251Q}$	MMFRDQVGVLAGWFKGWNECEQTVALLSLLKRVSQTQARFLQLCL<br>EHSLADCAELHVLEGEANSPGIINQWQQESKDKVISLLLTHLPLLKPG<br>NLDAKAEYMKLLPKILAHSIEHNQHIEESRQLLSYALIHPATSLEDRSA<br>LAMWLNHLEDRTSTSFGSQNRGRSDSVDYGQTHYYHQRQNSDDK<br>LNGWQNSRDSGICISASNWQDKSLGCENGHVPLYSSSSVPATINTIG<br>TGASTNVPAWLASLALHQYAALFSQMTYEEMMALTECQLEAQNVTK<br>GARHKIVISIQKLKERQNLLKSLERDIIEGGSLRTPLQELHQMILTPIKA<br>YSSPSTTPEVRCREPSLMESPSPDCKDSAAAVTSATASASAGASGG<br>LQPPQLSSCDGELAVAPLPEGDLPGQFTRVMGKVCTQLLVSRPDEE<br>NISSYLQLLDKCLVHEAFTETQKKRLLSWKQQVQKLFRSFPRKTLLDI<br>SGYRQQRNRGFGQSNSLPTASSVGSGMGRRNPRQYQIASRNVPS<br>ARLGLLGTSGFVSSNQRHTAANPTIMKQGRQNLWFANPGGSNSMP<br>SRTHSSVQKTRSLPVHTSPQNMLMFQQPEFQLPVTEPDINNRLESL<br>CLSMTEHALGDGVDRTSTI
SAMD4^{A281Q}	MMFRDQVGVLAGWFKGWNECEQTVALLSLLKRVSQTQARFLQLCL<br>EHSLADCAELHVLEGEANSPGIINQWQQESKDKVISLLLTHLPLLKPG<br>NLDAKAEYMKLLPKILAHSIEHNQHIEESRQLLSYALIHPATSLEDRSA

	LAMWLNHLEDRTSTSFGSQNRGRSDSVDYGQTHYYHQRQNSDDK<br>LNGWQNSRDSGICISASNWQDKSLGCENGHVPLYSSSSVPATINTIG<br>TGASTNVPAWLKSLRLHKYAALFSQMTYEEMMALTECQLEAQNVTK<br>GQRHKIVISIQKLKERQNLLKSLERDIIEGGSLRTPLQELHQMILTPIKA<br>YSSPSTTPEVRCREPSLMESPSPDCKDSAAAVTSATASASAGASGG<br>LQPPQLSSCDGELAVAPLPEGDLPGQFTRVMGKVCTQLLVSRPDEE<br>NISSYLQLLDKCLVHEAFTETQKKRLLSWKQQVQKLFRSFPRKTLLDI<br>SGYRQQRNRGFGQSNSLPTASSVGSGMGRRNPRQYQIASRNVPS<br>ARLGLLGTSGFVSSNQRHTAANPTIMKQGRQNLWFANPGGSNSMP<br>SRTHSSVQKTRSLPVHTSPQNMLMFQQPEFQLPVTEPDINNRLESL<br>CLSMTEHALGDGVDRTSTI
**SAMD4^{H86P}**	MMFRDQVGVLAGWFKGWNECEQTVALLSLLKRVSQTQARFLQLCL<br>EHSLADCAELHVLEGEANSPGIINQWQQESKDKVISLLLTPLPLLKPG<br>NLDAKAEYMKLLPKILAHSIEHNQHIEESRQLLSYALIHPATSLEDRSA<br>LAMWLNHLEDRTSTSFGSQNRGRSDSVDYGQTHYYHQRQNSDDK<br>LNGWQNSRDSGICISASNWQDKSLGCENGHVPLYSSSSVPATINTIG<br>TGASTNGGSLRTPLQELHQMILTPIKAYSSPSTTPEVRCREPSLMES<br>PSPDCKDSAAAVTSATASASAGASGGLQPPQLSSCDGELAVAPLPE<br>GDLPGQFTRVMGKVCTQLLVSRPDEENISSYLQLLDKCLVHEAFTET<br>QKKRLLSWKQQVQKLFRSFPRKTLLDISGYRQQRNRGFGQSNSLPT<br>ASSVGSGMGRRNPRQYQIASRNVPSARLGLLGTSGFVSSNQRHTA<br>ANPTIMKQGRQNLWFANPGGSNSMPSRTHSSVQKTRSLPVHTSPQ<br>NMLMFQQPEFQLPVTEPDINNRLESLCLSMTEHALGDGVDRTSTI-

Supplementary Table S2. Genes enriched in Flag IP.

chr	Gene	Reads in IP	Reads in IgG	p-value	Fold Change	Gene and enrich peak overlapped base
chr4	Errfi1	77	0	1.3E-23	77	228
chr2	Cebpb	72	0	4.2E-22	72	343
chr19	Fads1	53	0	2.2E-16	53	361
chr11	Timp2	48	0	7.1E-15	48	224
chr17	Tnfrsf12a	42	0	4.5E-13	42	293
chr10	Bsg	39	0	3.6E-12	39	93
chr16	Hmgn1	39	0	3.6E-12	39	140
chr4	Ece1	36	0	2.9E-11	36	87
chr10	Ncln	34	0	1.2E-10	34	273
chr17	Stub1	34	0	1.2E-10	34	271
chr10	Cited2	32	0	4.7E-10	32	226
chr7	Iqgap1	31	0	9.3E-10	31	162
chr6	Mlf2	30	0	1.9E-09	30	189
chr8	Isyna1	29	0	3.7E-09	29	253
chr1	Myo1b	25	0	6.0E-08	25	168
chr5	Phkg1	25	0	6.0E-08	25	162
chr9	Ctnnb1	24	0	1.2E-07	24	160
chr7	Fbl	23	0	2.4E-07	23	136
chr13	Pdlim7	23	0	2.4E-07	23	210
chr6	Immt	22	0	4.8E-07	22	177
chr14	Ipo4	22	0	4.8E-07	22	135
chr10	Ppa1	21	0	9.5E-07	21	160
chr8	Col4a2	20	0	1.9E-06	20	147
chr3	Gmps	19	0	3.8E-06	19	158
chr5	Denr	18	0	7.6E-06	18	111
chr15	Cyc1	16	0	3.1E-05	16	140
chr17	Ddr1	16	0	3.1E-05	16	152

chr4	Ptprf	16	0	3.1E-05	16	80
chr10	Tcf3	16	0	3.1E-05	16	128
chr4	Dvl1	15	0	6.1E-05	15	68
chr6	Epha1	15	0	6.1E-05	15	124
chr11	Hdac5	15	0	6.1E-05	15	145
chr4	Megf6	15	0	6.1E-05	15	135
